# Strontium Oxide Deposited onto a Load-Bearable and Porous Titanium Matrix as Dynamic and High-Surface-Contact-Area Catalysis for Transesterification

**DOI:** 10.3390/nano8120973

**Published:** 2018-11-26

**Authors:** Han Lee, Jiunn-Der Liao, Mu Hsuan Lee, Bernard Haochih Liu, Wei-En Fu, Kundan Sivashanmugan, Yung-Der Juang

**Affiliations:** 1 Department of Materials Science and Engineering, National Cheng Kung University, 1 University Road, Tainan 701, Taiwan; rick594007@mail.ncku.edu.tw (H.L.); N56055021@mail.ncku.edu.tw (M.H.L.); hcliu@mail.ncku.edu.tw (B.H.L.); N58991304@mail.ncku.edu.tw (K.S.); 2 Medical Device Innovation Center, National Cheng Kung University, 1 University Road, Tainan 701, Taiwan; 3 Center for Micro/Nanoscience and Technology, National Cheng Kung University, Tainan 70101, Taiwan; 4 Center for Measurement Standards, Industrial Technology Research Institute, No. 321, Kuang Fu Road, Sec. 2, Hsinchu 300, Taiwan; WeienFu@itri.org.tw; 5 Department of Materials Science, National University of Tainan, Tainan 700, Taiwan; juang@mail.nutn.edu.tw

**Keywords:** Strontium oxide, porous titanium scaffold, transesterification, biodiesel

## Abstract

Strontium oxide (SrO) deposited onto a porous titanium (Ti)-based scaffold (P-Ti) is a promising and novel approach for high-throughput transesterification. Notably, a highly porous and calcinated scaffold provides a load-bearable support for a continuous process, while the calcinated SrO catalyst, as it is well distributed inside the porous matrix, can extend its surface contact area with the reactant. In this work, the formation of transesterification reaction with the conversion and production of olive oil to biodiesel inside the porous matrix is particularly examined. The as-designed SrO-coated porous titanium (Ti)-based scaffold with 55% porosity was prepared via a hydrothermal procedure, followed by a dip coating method. Mechanical tests of samples were conducted by a nanoindentator, whereas the physical and chemical structures were identified by IR and Raman Spectroscopies. The results implied that SrO catalysts can be firmly deposited onto a load-bearable, highly porous matrix and play an effective role for the transesterification reaction with the oil mass. It is promising to be employed as a load-bearable support for a continuous transesterification process, such as a process for batch or continuous biodiesel production, under an efficient heating source by a focused microwave system.

## 1. Introduction

Nowadays, the development of industries as well as the demand toward a high living standard have increased the consumption of energy and the use of petroleum/fuel cells. As a consequence, air pollution and global warming problems are a concern and mainly directed to the emission of CO_2_. For countries lacking energies, for their industrial development, the increased use of the imported petroleum leads to intensifying and localizing the extent of air pollution, mainly caused by the emission of CO_2_ and, as a consequence, to magnifying the global warming problem. Many extensive negotiations such as the Kyoto Protocol [1] have been conducted and established to protect our living environment and to make it sustainable. Hence, it is an urgent issue to find out solutions to reduce the consumption of energy derived from fossil fuel and, at the same time, to replace a part of it through the conversion of green energy [2]. For example, such a solution can be found by using biodiesel fuel. Unlike petroleum and other kinds of fossil fuels, it is almost biodegradable and friendly to the environment.

Nowadays, there are increasing numbers of countries encouraging the use of biodiesel to have the unprecedented potentiality for endurable energy application in the future [3]. Currently, there are some effective methods [4] that have been widely applied to the generation of biodiesel resources. For example, straight use and a mixture of raw oil, thermal cracking, microemulsions, and transesterification are applied [4]. Straight use and blending of raw oil are based on a condition of liquid nature portability, and its heat content is around 80% that of diesel fuel, which is relatively spectacular [5]; it is readily available and renewable [6]. However, because the method easily deteriorates and loses its volatility, it is difficult to consider for a practical use [7]. In addition, reacting with unsaturated hydrocarbon chains is regarded as an unfavorable phenomenon [8]. In regard to engine applications, it may cause choking, trumpet formation, or even carbon deposits [8]. Furthermore, oil ring sticking, thickening, and solidifying of the lubricating oil are the factors that make this method lack feasibility [9]. Microemulsions are considered to be a colloidal balance diffusion of an optically isotropic fluid microstructure with a size usually in the range of 1~150 nm. It forms naturally from two kinds of immiscible fluids and one or even more ionic or nonionic amphiphile substances [10]. It also features a favorable spray arrangement while in combustion and has relatively lower fuel viscosity, compared with the average fuel. Nevertheless, an insufficient cetane number and energy content make it unsuitable for the application [11].

Transesterification mainly works as the interaction between a solid fat or liquid oil and an alcohol under a catalyst used to generate glycerol and esters. There are many aspects to take into consideration, such as time consumption during the reaction, the quality of the product, or the necessity for post-treatment. Generally, alkaline compounds are ordinarily seen in terms of homogeneous catalysts in the biodiesel industry (e.g., sodium hydroxide and potassium hydroxide). Sodium hydroxide and potassium hydroxide catalysts have a characteristic of good solubility for use as biodiesel fuel and their common byproduct, glycerin, makes them difficult to purify and lowers their practical efficiency. The utilization of heterogeneous catalysts is a superb idea to make up for the demerits of homogeneous catalysts. Besides, they perform much better in microwave-promoted transesterification reactions. Strontium oxide (SrO) is superior to other catalysts in terms of conversion and reaction time, but at the same time [12], it has a significant disadvantage: Since SrO remains in the biodiesel after the reaction, separation of the catalyst and the product requires neutralization washing, resulting in a large amount of industrial wastewater pollution to the environment.

In our previous study, porous Ti with different forms or ratios of porosity has been fabricated using a hydrothermal procedure by the adjustment of NaCl content in Ti powder [13]. Hydrothermal method has been applied for producing a porous Ti-based scaffold at high temperature and high pressure with the inclusion of a removable pore former [13]. Besides, with the increasing demand for complex designs (e.g., a catalytic surface on/in the porous structure/shape), a catalyst coating can be infused [14]. In particular, SrO as one of the catalyst materials has gained a lot of attraction because of its capability to enhance many chemical reactions (e.g., Tishchenko reactions, nitroaldol reactions, and oxidative coupling of methane [15,16]). The combination of an appropriate Ti-based scaffold with a catalyst coating, therefore, may offer several advantages for designing a load-bearing catalytic scaffold. The sol–gel dip coating technique is typically applied for silt up a relatively large or irregularly shaped surface at low cost [14,17]. In this study, SrO solution can be sufficiently introduced into the porous structure and improve its superiority. With a SrO thin film coated on a porous Ti scaffold, the Ti-based porous SrO catalyst will be integrated for the transesterification reaction. Its mechanical and chemical properties will be characterized and discussed in this study.

## 2. Experimental Section

### 2.1. Preparation of P-Ti and Sr_x_-P-Ti_55

A Ti sample with 55 wt% addition of NaCl was prepared as shown in Figure S1 in Supporting Data (SD). The as-sintered porous (i.e., after NaCl removal) Ti sample is denoted as P-Ti_55. The chosen porosity of P-Ti_55 has been previously studied [14]. The sample was stored for further studies.

**Figure 1:**
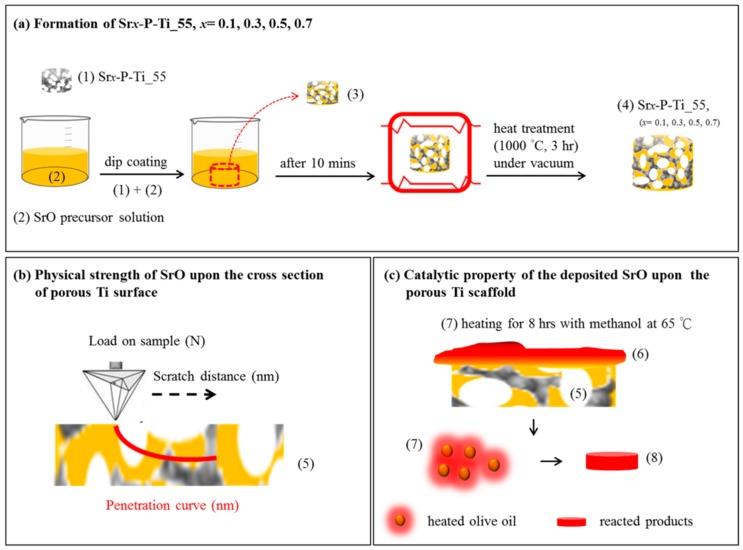
Schema for the formations of (**a**) SrO deposited upon a porous Ti scaffold (P-Ti): (1) the preparation of Sr*_x_*-P-Ti_55 (*x* = 0.1, 0.3, 0.5, 0.7); (2) SrO precursor solution; (3) the samples after removing (1) from (2); (4) the sample (3) after heat treatment (Sr*_x_*-P-Ti_55, the sample (4)). (**b**) The stiffness or adhesion test of SrO upon the cross-sectioned Sr*_x_*-P-Ti_55 (the sample (5)), followed by a physical strength test using a nano-scratch tip. (**c**) Evaluation of the deposited SrO as a catalyst for transesterification: (6) on the sample (5), depositing a very thin layer of olive oil (the sample (6)); (7) the sample (6) heating for 8 h at 65 °C, and taking from the top layers, which contains the transesterified products, SrO catalysts, and a small part of the sample (5); (8) smashing the sample (7) into powder, and then pressing it into a thin disc.

A thin-film SrO coating was deposited on P-Ti_55 through a dip coating procedure. Specifically, alcohol/Sr complexions were prepared by mixing strontium nitrate (Sr(NO_3_)_2_, 99%, Aldrich, St. Louis, MO, USA) and ethylene glycol ((CH_2_OH)_2_, 99.8%, Sigma, Cream Ridge, NJ, USA) with 0.1, 0.3, 0.5, and 0.7 M metal precursors. Subsequently, 100 μL of citric acid (C_6_H_8_O_7_) was added for every 1 mL of the as-prepared solution. These mixed solutions were used for the thin-film coating. The solution was moved for dip coating on the specimen at a speed of 50 mm/min. The as-coated P-Ti_55 was dried at 80 °C for 10 min and then heated at 65 °C for 8 h to obtain an adherent coating. Note that the heating temperature (<76 °C) and time (>6 h) are chosen to complete an esterification reaction [18]. The above procedures were illustrated in Figure 1a. The SrO-coated Ti samples for this study are denoted as Sr*_x_*-P-Ti_55, where *x* = 0, 0.1, 0.3, 0.5, and 0.7.

### 2.2. Quality Assessment of P-Ti_55 and Sr_x_-P-Ti_55

The crystalline structures of Sr_x_-P-Ti_55 were determined using X-ray diffraction (XRD, MiniFlex II, Rigaku, Tokyo, Japan); XRD patterns were obtained using CuKα radiation. Surface morphologies of Sr*_x_*-P-Ti_55 were examined using a Field-Emission Scanning Electron Microscope (FE-SEM, JSM-7001, JEOL, Tokyo, Japan). The resulting porosity was measured using *Archimedes* method with the distilled water.

### 2.3. Scratch Test and Catalytic Evaluation of the Coated SrO

The adherence property of SrO on P-Ti_55 was examined by a nanoindentor with the continuous stiffness measurement (Nano Indenter G200, MTS, Huntsville, AL, USA), which produces highly sensitive load-displacement data at the surface contact level. In the examination, the triangular pyramid tip of a *Berkovich* diamond indenter with a radius of 20 nm was used under a controlled relative humidity of 45% at 22 °C. *Poisson*’s ratio for Sr*_x_*-P-Ti_55 was set to 0.30. The loading process was controlled to have a surface approach velocity of 1 nm/s with a sensitivity of 5%. Nanohardness and stiffness measurements by the loading process were controlled to have a surface approach velocity of 1 nm/s with a sensitivity of 5%. A constant strain rate of 0.05/s at a chosen frequency of 75 Hz was applied. The calculation of nanohardness was mainly based on the *Oliver and Pharr* method [19,20]. For each sample, six different unbiased locations were chosen.

In addition, a scratch rate of 2 μm/s with an applied normal force increased from 0.01 to 50 mN was used. A maximum load of 50 mN and a scratch distance of 2000 μm were applied to reduce the load-increasing rate and to obtain a critical load (*L*_C_). For a given coating/substrate system, one or more critical scratch loads (*L*_CN_) were defined for progressive levels of damage on Sr*_x_*-P-Ti_55. Such examination is illustrated in Figure 1b.

In Figure 1c, the uses of the coated SrO as a catalyst for transesterification are illustrated. On the P-Ti_55 (5), a very thin layer (100~300 nm) of olive oil (6) (Taiwan Sugar Corp., Tainan City, Taiwan), purchased from a local supermarket, was deposited and followed by heating for 8 h with methanol at 65 °C (7). To analyze the product after transesterification, the powder was taken from the top layers of (7), which contained the heated interface between a very thin layer of olive oil and a small part of (5). The taken layers were smashed into powder, and then pressed into a thin disk (8). The thin discs Sr*_x_*-P-Ti_55, *x* = 0.5 and 0.7 with added olive oil droplet are denoted as *x* = 0.5 and *x* = 0.7, respectively. For the *x* = 0.5 and *x* = 0.7, followed by heating, they are denoted as *x* = 0.5* and *x* = 0.7*, respectively. The *x* = 0.5* and *x* = 0.7* were prepared for studying the formation of transesterification reaction.

### 2.4. Surface Analysis on the Thin Disc Sr_x_-P-Ti_55

From the basic study of transesterification, SrO powder was mixed with olive oil; the mixture was then analyzed by Differential Scanning Calorimeter (DSC, TA Instruments DSC 2010, New Castle, DE, USA). The heating temperatures were increased from 25 to 100 °C at a rate of 5 °C/min.

The thin disc *x* = 0.5* and *x* = 0.7* were employed for subsequent chemical analysis on the surfaces. Infrared spectra of the thin-disc samples were obtained using an Attenuated Total Reflection–Fourier Transform Infrared spectrometer (ATR-FTIR, Thermo/Nicolet, Waltham, MA, USA) in the wavenumber range of 3800–600 cm^−1^ with 2 cm^−1^ resolution and 6 scans at room temperature. Raman spectra were obtained using a Raman spectrometer with a confocal microscope (Renishaw, Gloucestershire, UK). He–Ne and diode lasers with an excitation wavelength of 785 nm were respectively applied. An air-cooled CCD was used as the detector and the incident power was about 3 mW. The thin-disc samples were scanned with an exposure time of 10 s over an area of 1 μm × 1 μm, using a 50× objective. Ten consecutive measurements on different samples were averaged. All spectra were normalized using the peak fit software. Both spectra were examined to disclose the formation of esterification reaction over the thin disc *x* = 0.5* and *x* = 0.7*.

By measuring the binding energies (BEs) of elements using X-ray Photoelectron Spectroscopy (XPS, PHI 5000, VersaProbe, Kanagawa, Japan), chemical bonds on the surfaces of *x* = 0.5* and *x* = 0.7* were characterized. The functionalities of constituent elements (i.e., Sr 3d and O 1s for the catalyst SrO, C 1s, and O 1s for the reactants and products) were curve-fitted using the software *Origin* and related data information in order to clarify the formation of the esterification reaction.

## 3. Results and Discussion

### 3.1. Physical Properties of the Cross-Sectioned Surface of Sr_x_-P-Ti_55

**Figure 2:**
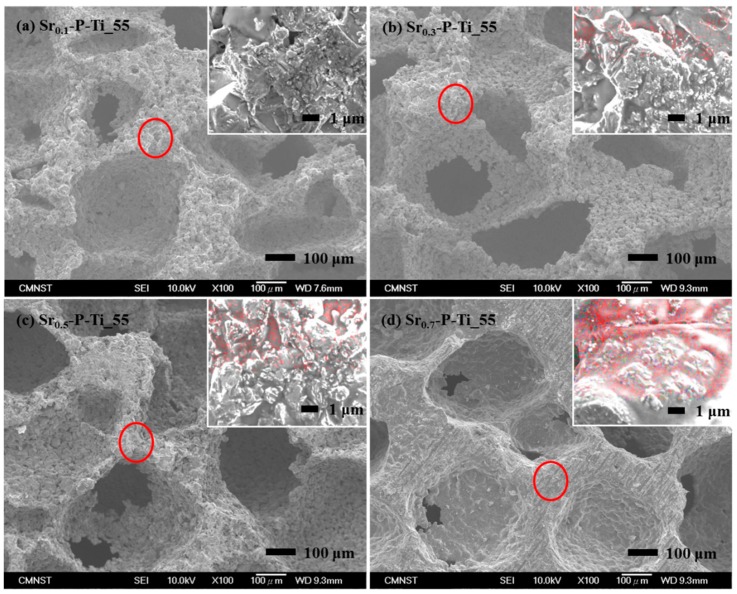
SEM morphologies from the cross-sectioned surfaces of Sr*_x_*-P_Ti_55, *x* = (**a**) 0.1, (**b**) 0.3, (**c**) 0.5, and (**d**) 0.7. Their top-right images were taken from EDS element mapping; the red color is the element Sr.

Surface morphologies of cross-sectioned Sr*_x_*-P-Ti_55 are shown in Figure 2a–d. The circled parts were respectively enlarged on the top-right photo images. The distributions of Sr element within a nonporous mapping area were measured and are shown in red points. The top-right photo images show that SrO in different concentrations was competent to penetrate into P-Ti_55, to remain in the porous matrix, and to correlate with the increase of SrO concentrations (from 0.1 to 0.7 M). As shown in Figure 2a–d, the photo images showed the presence of interconnected pores in the cross-sectioned P-Ti_55, which remained mostly unchanged as the concentration of SrO was increased. Therefore, the current method to deposit SrO in the porous matrix of P-Ti_55 does not alter its pore size and dimension.

**Figure 3:**
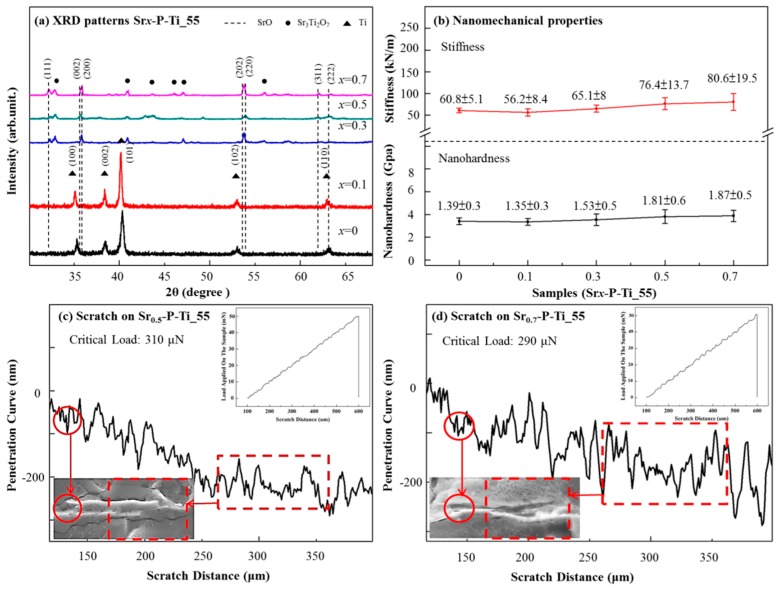
(**a**) XRD patterns for Sr*_x_*-P-Ti_55 with various SrO concentrations; the samples were annealed at 1000 °C for 3 h. Variations in (**b**) nanohardness and stiffness versus load on Sr*_x_*-P-Ti_55 samples were estimated at selected locations. Scratch test results for surfaces of (**c**) Sr_0.5_-P_Ti_55 and (**d**) Sr_0.7_-P_Ti_55, in which the inner figures demonstrate the scratch distance versus applied load curves for the representative samples.

In Figure 3a, XRD patterns of Sr*_x_*-P-Ti_55 showed that the polycrystalline structure of SrO depends on the metal precursor’s concentration at 1000 °C [21,22]. Two structures, tetragonal (t)-SrO (JCPDS card No. 27-1304) [23] and (t)-Sr_3_Ti_2_O_7_ (JCPDS card No. 11-0663) clearly appeared for Sr_0.7_-P-Ti_55. Note that XRD patterns show reflection peaks of tetragonal SrO in the 2θ range of 30 to 65°, corresponding to (111), (002), (200), (202), (220), (311), and (222). For example, for Sr_0.1_-P-Ti_55, the peaks at 2θ = 30° and ~68° were respectively assigned to (100), (002), (101), (102), and (110) reflections of tetragonal Ti (JCPDS card No. 44-1294) [24]. At higher concentrations of SrO (e.g., 0.5 and 0.7), a second-phase pattern appeared with reflection peaks of tetragonal Sr_3_Ti_2_O_7_ in the 2θ range of 33° to 56°, corresponding to (112), (116), (0010), (200), (118), and (1110). The presence of the second phase occurring at 1000 °C, under atmospheric condition, reveals the formation of a Sr-Ti-O compound, whereas the mechanical property of SrO upon Ti/TiO_2_ and the remaining catalytic property of SrO are ambiguous. The thickness of the calcinated SrO film was calculated using XRD patterns and the Scherrer formula, as 25.9 and 43.1 nm for Sr_0.5_-P-Ti_55 and Sr_0.7_-P-Ti_55, respectively (see Table S1 in the Supporting data).

### 3.2. Mechanical Properties of the Cross-Sectioned Surfaces of Sr_x_-P-Ti_55

Figure 3b shows the variations of nanohardness and stiffness as a function of the load on the selected area of Sr*_x_*-P-Ti_55. Taking the surface of Sr*_x_*-P-Ti_55, *x* = 0, as the reference, the nanohardness of Sr*_x_*-P-Ti_55, *x* = 0, 0.1, 0.3, and 0.5, were 1.39 ± 0.3, 1.35 ± 0.3, 1.53 ± 0.5, and 1.81 ± 0.6 GPa, respectively. No obvious change was found. As the SrO concentration increased to Sr_x_-P-Ti_55, *x* = 0.7 (1.87 ± 0.5 GPa), its nanohardness at the cross-sectioned surface slightly increased. A similar trend was obtained for the stiffness measurement of Sr*_x_*-P-Ti_55, *x* = 0, 0.1, 0.3, and 0.5. The values were 60.8 ± 5.1, 56.2 ± 8.4, 65.1 ± 8, and 76.4 ± 13.7 GPa, respectively. No obvious change was found. As the SrO concentration increased to Sr_x_-P-Ti_55, *x* = 0.7 (80.6 ± 19.5 GPa), its stiffness at the cross-sectioned surface also slightly increased. A slight increase in nanohardness or stiffness on the cross-sectioned Sr*_x_*-P-Ti_55, *x* = 0.5 and 0.7, is most probably due to the formation of a secondary phase (e.g., Sr_3_Ti_2_O_7_) at the interface of SrO upon P-Ti_55. Since the base material, porous Ti substance, remains the same, therefore, their normal load-displacement measurements did not cause significant changes.

The cohesive and adhesive forces based on the measured critical load (*L*c) in the SrO coating and the undulate depths at the P-Ti_55 interface were studied using scratch tests [25]. In Figure 3c,d, because the surface property of Sr*_x_*-P-Ti_55 is different from that of P-Ti_55, its inclination of the undulate curve may change accordingly. The Lc values for Sr_0.5_-P-Ti_55 and Sr_0.7_-P-Ti_55 were particularly measured. Based on the first significant decrease in their penetration depths, the Lc values for Sr_0.5_-P-Ti_55 and Sr_0.7_-P-Ti_55 were calculated as 310 and 290 µN, respectively. To determine the adhesion forces between the thin-film SrO and P-Ti_55, the scratch distances with respect to the surface properties (e.g., the coated thickness and roughness of both materials) of the cross-sectioned Sr_0.5_-P-Ti_55 and Sr_0.7_-P-Ti_55 were measured as 190 to 210 nm, respectively. The results indicate that the cross-sectioned surface of Sr_0.5_-P-Ti_55 has a relatively high value of *L*c and the scratch tip shifts a relatively short distance. Thus, the cross-sectioned surface of Sr_0.5_-P-Ti_55 creates a stiffer interface.

By taking the penetration curve at a depth of 250 nm with a scratch distance of 250 µm, the adhesion force values for Sr_0.5_-P-Ti_55 and Sr_0.7_-P-Ti_55 were measured around 4.82 and 4.78 mN, respectively. From the Lc measurements, the cross-sectioned surface of Sr_0.5_-P-Ti_55 creates a much stiffer interface than that of Sr_0.7_-P-Ti_55. On the bottom-left SEM images in Figure 3c,d, the scratched surfaces were shown by circling the scratched regions, in which, without applying the scratch test, such infinitesimal change owing to the coverage of SrO particles on the flat surfaces of P-Ti_55 was difficult to be distinguished.

### 3.3. Chemical Analysis on the x = 0.5* and x = 0.7*

**Figure 4:**
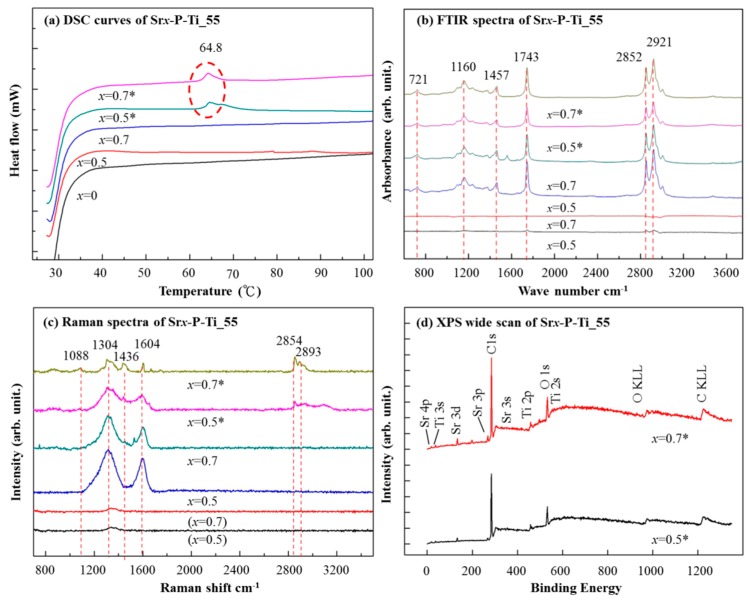
(**a**) DSC curves obtained from the samples Sr*_x_*-P-Ti_55 (x = 0, 0.1, 0.3, 0.5, and 0.7). (**b**) The characteristic peaks obtained from FTIR Spectroscopy for the biodiesel at 721, 1160, 1457, 1743, 2852, and 2921 cm^−1^. Note that *x* = 0.5 and *x* = 0.7 indicate the addition of olive oil without heating. (**c**) The characteristic peaks obtained from Raman Spectroscopy for the biodiesel at 1088, 1304, 1436, 1604, 2854, and 2893 cm^−1^. (**d**) Wide scan XPS spectra for Sr*_x_*-P-Ti_55 (*x* = 0.5 and 0.7). Detailed information is discussed in the text.

At first, the DSC analysis was performed in the temperature range from RT to 100 °C for testing the occurrence of the catalytic reaction. In Figure 4a, an exothermic peak at 64.8 °C [26] appeared as a little olive oil interacting with SrO–Ti powder (i.e., Figure 1c (7)). The exothermic reaction is correlated with the decomposition of reactants such as mono-alkyl esters of saturated or unsaturated long-chain fatty acid into products such as an oxidized product involving the long carbon chain [27].

In Figure 4b, in the methanolysis of olive oil, FTIR spectra of reactants and products are very similar, owing to the high chemical similarity that exists among triglycerides and methyl esters [28], with the exception of small differences as reported by Zagonel et al. [29], for example, the peak 1743 cm^−1^ (C=O) to distinguish whether there is biodiesel functional group. In addition, the analysis of the FAME compound shows the signature of esters at 1742 cm^−1^ and also the presence of oleic acid double bonds at 3009 cm^−1^ (C=C) [29]. Therefore, similar peaks for *x* = 0.5* and *x* = 0.7* can be clearly observed in the following regions: (1) a broader region covering wave numbers from 700 to 1500 cm^−1^ (721, 1160, and 1457 cm^−1^), (2) a region around 1743 cm^−1^, including the stretching vibrations of carbonyl groups [29], and (3) a latter region (from 2600 to 3100 cm^−1^) readily attributing to the extent of glycerol substitution in fatty acids by methoxy radicals (methanolysis). Note that for the products, 90% of biodiesel and 10% of glycerol are respectively formed [30]. The broader region shown in (1) is more complex, which displays a series of overlapped signals that are likely to interfere with the development of the esterification reaction [28]. Presumably, Raman spectra of vegetable oil and its corresponding methyl ester may show several differences [31].

In Figure 4c, Raman spectra from *x* = 0.5* and *x* = 0.7* are shown. Based on the DSC analysis, an exothermic peak at 64.8 °C was found. For the sensitivity of temperature, a 785 nm Raman laser was chosen. For the thin discs *x* = 0.5 and *x* = 0.7, the characteristic peaks at 1304 and 1604 cm^−1^ were observed. For the thin discs *x* = 0.5* and *x* = 0.7*, the intensities of the peaks at 1304 and 1604 cm^−1^ were reduced, whereas the new peaks at 1088, 1436, 2854, and 2893 cm^−1^ were found. The peaks at 1088 and 1436 cm^−1^ were from nonconjugated out-of-plane symmetric bending modes (=C–H bond), while those at 2854 and 2893 cm^−1^ were related to asymmetric aliphatic CH [31,32]. These new Raman peaks usually correspond to the characteristic peaks of biodiesel [31,32]. With the result from DSC analysis and the presence of Raman-active modes, a transesterification reaction occurred upon the surfaces of the thin discs *x* = 0.5* and *x* = 0.7*. There exists the formation of O vacancies when depositing a thin film, which may thereafter affect its optical and electrical properties [33,34]. The O vacancies lead to structural imperfections that may play an important role in the transesterification process.

### 3.4. XPS Characterization on the Surfaces of the Discs x = 0.5* and x = 0.7*

**Figure 5:**
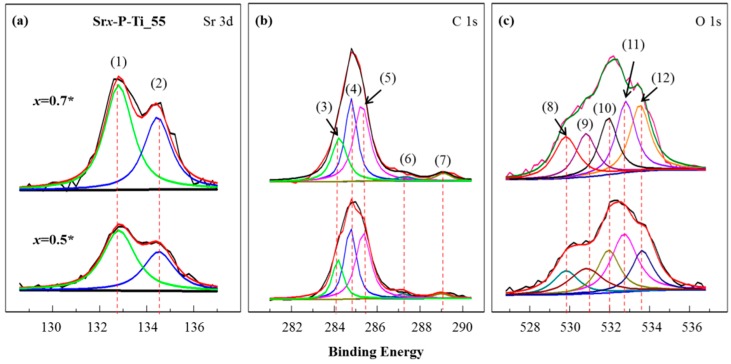
Curve-fitting data for XPS spectra of (**a**) Sr 3d, (**b**) C 1s, and (**c**) O 1s on *x* = 0.5* and *x* = 0.7*, respectively. The curve-fitted peaks are respectively assigned: (1) 132.9 eV, Sr 3d_5/2_, (2) 134.6 eV, Sr 3d_3/2_, (3) 284.2 eV, C=O, (4) 284.8 eV, C–C, (5) 285.5 eV, C–H, (6) 287.1 eV, C–O, (7) 289.2 eV, COO, (8) 529.8 eV, O-containing ions in SrO, (9) 531 eV, C-O, (10) 532 eV, loosely bound hydroxide groups, M–OH, (11) 532.8 eV, C=O, (12) at 533.7 eV, C–O.

To understand the chemical bonds on the thin discs *x* = 0.5* and *x* = 0.7*, an XPS study was performed. In Figure 4d, an XPS survey spectra showed the presence of Ti, Sr, C, and O elements’ functionalities. In Figure 5, further studies on the core levels of Sr, O, and C-associated BEs, namely, Sr 3d, C 1s, and O 1s spectra, were curve-fitted.

In Figure 5a, the peaks (1) at 132.9 eV (Sr 3d_5/2_) and (2) at 134.6 eV (Sr 3d3_/2_) with a comparable ratio for the thin discs *x* = 0.5* and *x* = 0.7*, are assigned to BEs of Sr with O, as summarized in Table 1. In Figure 5c, the peak (8) at 529.8 eV from the curve-fitted O 1s spectrum is assigned to O-containing ions in Sr–O [28]. The peak area ratio, O_peak(8)_/O_total_, indicates O lattice content and related defects. As summarized in Table 2, the ratio increased from 12.9 (*x* = 0.5*) to 24.0% (*x* = 0.7*), which shows a significant increase of SrO attached on P-Ti_55. The peak (10) corresponds to the formation of M–OH [35,36]. The O_peak(10)_/O_total_ ratio changed from 20.2 (*x* = 0.5*) to 24.4% (*x* = 0.7*), which shows the increased quantity of SrO hydration [26].

The C 1s spectrum in Figure 5b provides the information about the occurrence of the transesterification reaction. It was curve-fitted using a Gaussian distribution into five components: the peaks (3) at 284.2 eV (C=O), (4) at 284.8 eV (C–C), (5) at 285.5 eV (C–H), (6) at 287.1 eV (C–O, a methyl ester), and (7) at 289.2 eV (O=CO). As summarized in Table 1, the peaks (3), (6), and (7) from the curve-fitted C 1s spectrum in Figure 5b and the peaks (11) at 532.8 eV (C=O) and (12) at 533.7 eV (C–O) from the curve-fitted O 1s spectrum in Figure 5c correspond to the formation of biodiesel. For the products, since the quantity of added olive oil was the same for the thin discs *x* = 0.5* and *x* = 0.7*, as summarized in Table 2, the O_peak(11)_/O_total_ and O_peak(12)_/O_total_ ratios insignificantly changed from 17.5 (*x* = 0.5*) to 18.6% (*x* = 0.7*) and 17.8 (*x* = 0.5*) to 17.6% (*x* = 0.7*), respectively. From the semiqualitative analysis, XPS data has good agreement with the theoretical molecular composition of these organic species.

In addition, the peaks (9) at 531.0 eV and (12) at 533.7 eV are taken as the reference compound (–O–CH_3_) and associated with the C 1s contribution for the peaks (6) at 287.1 eV and (7) at 289.2 eV. The O_peak(9)_/O_total_ ratio decreased from 31.6 (*x* = 0.5*) to 15.4% (*x* = 0.7*), which indicates the result from biodiesel production and the change in the chemical structure due to a degradation process: most of these organic groups decomposed into CO_2_ and escaped from the system, but still some minor amount of the byproducts derived from decomposition and some carbon substitute atom might not have been totally eliminated. In addition, glycerol contains a large amount of -CH_2_ and -OH, and due to the bond energy difference between Sr–O, C–O, and C–C, bond cleavage under the influence of temperature could happen to C–O first [37,38].

### 3.5. Chemical Kinetics for the Transesterification Reaction

In this study, the cross-sectioned P-Ti_55, the quantity of olive oil droplet, and the process to have *x* = 0.5* and *x* = 0.7* are presumably constant. The variation may thus come from the amount of SrO on P-Ti_55.

**Figure 6:**
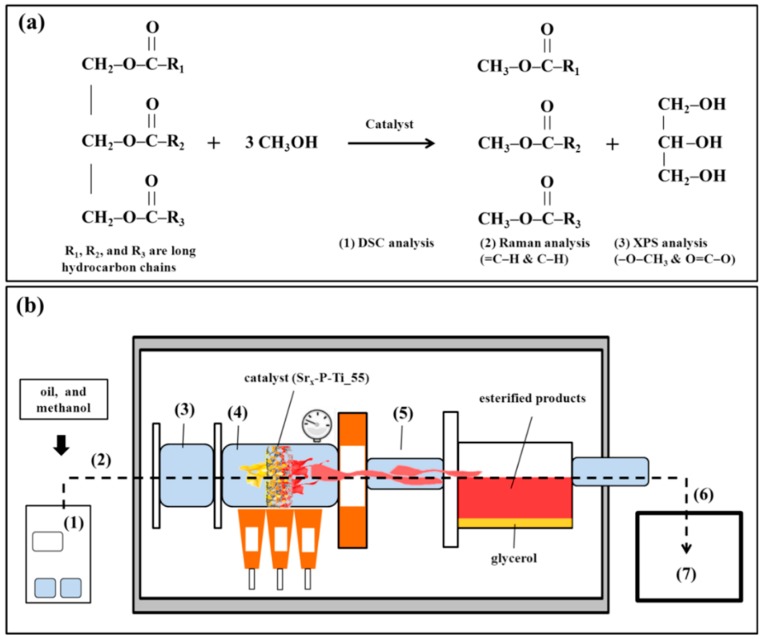
(**a**) The reactants and the products for the transesterification reaction of olive oil by SrO catalyst, which are respectively characterized by: (1) DSC, (2) Raman Spectroscopy, and (3) XPS. (**b**) A layout for a high-throughput transesterification process using Sr*_x_*-P-Ti_55 as a dynamic and load-bearable support: (1) mixing oil with methanol by a magnetic stirrer, (2) entering the treatment system through a pipe, (3) sucking up the mixed solution by a feeding pump, then flowing olive oil with a penetrating speed into the Sr_0.5_-P-Ti_55 matrix, (4) heating the Sr_0.5_-P-Ti_55 matrix with olive oil in a close chamber with, for example, a focused microwave heating source, (5) collecting the products and residual substances in a chamber, and (6) separating the biodiesel into a tank (7).

In Figure 6a, the transesterification reaction or biodiesel conversion has been identified by DSC, Raman-active modes, and XPS data. In particular, a semiquantitative measurement was realized by XPS study, which demonstrated the changes from the curve-fitted peaks of Sr 3d, C 1s, and O 1s spectra. The endothermic reaction and the investigation of chemical bonds in the reactants and products are summarized in Figure 6a. For the reactants, triglycerides is a kind of organic compound allied to the derivation from animal fat or plant oil that is composed of an ester containing the glycerol and three free fatty acids. The alcohol is deprotonated with a base to make nucleophile tougher in the transesterification procedure [39]. Despite natural variations (e.g., those caused by the degradation of the SrO catalyst and the quality of olive oil), the conversion process of olive oil into biodiesel and the reaction rate are mainly based on the formation of the transesterification reaction [12]. SrO catalyst lowers the activation energy for the transesterification reaction, which promotes the decomposition of ester bonds and the formation of a tetrahedral intermediate substance. However, the degradation of SrO catalysts lowers the biodiesel conversion rate. Factors that may affect the reaction include the reaction temperature and the evaporation of methanol, which will also be varied with the oil:methanol ratio [40,41,42]. Previous studies have shown that the optimal oil-to-methanol ratio is about 1:6 [12]. Other ratios such as 1:4 and 1:8 have been studied. Nevertheless, lower mean yields compared to that of 1:6 resulted [12,43]. Processing parameters such as the fluid flow velocity, which may reduce the clustering effect of reactants and soap formation, and energy efficiency, which may realize a temperature control, are of importance for carrying out the scale-up process [12]. In Figure 6b, we illustrate a practical use of Sr_0.5_-P-Ti_55 in a continuous process for biodiesel conversion and production. With the porosity and considerable mechanical strength of P-Ti_55 and appropriate amount of SrO bonded with Ti-based matrix, an appropriate oil flowing speed penetrating into the porous matrix and reaction with the firmly attached SrO is feasible [13,14]. The Sr_0.5_-P-Ti_55 is thus promising to be employed in a continuous process for biodiesel conversion and production [12].

## 4. Conclusions

In this study, a highly porous Ti-based scaffold (55% porosity, P-Ti_55) with considerable strength is particularly employed for conducting a load-bearable application. Strontium oxide (SrO) as the catalyst is spin-coated into the porous matrix and calcinated with Ti-based scaffold (i.e., Sr*_x_*-P-Ti_55, *x* = 0.5 or 0.7 for this study). Then, a firmly deposited Sr–Ti–O compound is created. In order to assess the occurrence of the endothermic reaction, Sr*_x_*-P-Ti_55 is cross-sectioned and heated with olive oil on the surfaces. The thin-film SrO reacted with the olive oil with top-layer Ti is accordingly removed, and then pressed into a disc (*x* = 0.5* or *x* = 0.7*). By examining the *x* = 0.5* and *x* = 0.7*, the chemical structure of biodiesel and the role of catalyst are identified by IR and Raman Spectroscopies and XPS curve-fitting analysis. The formation of the esterification reaction and the production of biodiesel are successfully examined and assisted through the presence of firmly-attached SrO catalyst inside the porous Ti-based matrix. It brings about a load-bearable condition for the flowing oily mass and contributes to a continuous treating process for biodiesel conversion and production. In future work, the applied parameters, including the oil-to-methanol ratio, microwave heating power, required reaction temperature, and loading quantity of the catalyst are firstly optimized in the batch unit. Afterwards, the adjusted flowing rate will be particularly studied in the continuous fluid flow system with a focused microwave heating device.

## Figures and Tables

**Table 1:** The binding energies and related peaks (deconvoluted) for the chemical states of Sr, C, and O elements.

Samples	Peak	Bond	Bonding Energy (eV)
Sr*_x_*-P_Ti_55 (*x* = 0.5*, and 0.7*)	Sr 3d	(1) Sr with O ^a^	132.9
		(2) Sr with O ^a^	134.6
	C 1s	(3) C=O ^b^	284.2
		(4) C–C	284.8
		(5) C–H	285.5
		(6) C–O ^b^	287.1
		(7) COO ^b^	289.2
	O 1s	(8) O-containing ions in SrO ^a^	529.8
		(9) C–O	531.0
		(10) M–OH	532.0
		(11) C=O ^b^	532.8
		(12) C–O ^b^	533.7

^a^ SrO: related peaks (1), (2), (8), and (10); ^b^ Biodiesel: related peaks (3), (6), (7), (11), and (12).

**Table 2:** Taking the area ratios of O-related peaks (deconvoluted) for the changes of SrO (in proportion to the quantity) and biodiesel (one of the products). Note that the area of oxygen peak = y, Increase = Inc., Decrease = Dec.

Sr_x_-P_Ti_55	*x* = 0.5* with Oil					*x* = 0.7* with Oil				
	Sr–O	C–O	M–OH	C=O	C–O	Sr–O	C–O	M–OH	C=O	C–O
BE (eV)	529.8	531.0	532.0	532.8	533.7	529.8	531	532	532.8	533.7
Peak area ratio (O_peak(y)_/O_total_%)	12.9 *y* = 8	31.6 *y* = 9	20.2 *y* = 10	17.5 *y* = 11	17.8 *y* = 12	24 *y* = 8	15.4 *y* = 9	24.4 *y* = 10	18.6 *y* = 11	17.6 *y* = 12
Compare (_0.5* and 0.7*_)						Inc.	Dec.	Inc.	Inc.	Dec.

## Supplementary Materials

The following are available online at http://www.mdpi.com/2079-4991/8/12/973/s1, Figure S1: Fabrication processes: porous Ti samples are obtained by mixing Ti powder with NaCl, and followed by the hydrothermal technique, which may thereafter remove NaCl from the Ti matrix, leave the solid pore sites, and form porous structures, Table S1: Calculation of grain size by using XRD.
