# Supplementary material for: Strontium Oxide Deposited onto a Load-Bearable and Porous Titanium Matrix as Dynamic and High-Surface-Contact-Area Catalysis for Transesterification
Source: Nanomaterials (Basel). 2018 Nov 26;8(12):973. doi: 10.3390/nano8120973 (PMC6315904; doi:10.3390/nano8120973)
Supplement: Supplementary file 1 [file nanomaterials-08-00973-s001.pdf]

Supporting Data

Strontium Oxide Deposited onto a Load-Bearable and Porous Titanium Matrix as Dynamic and High-Surface-Contact-Area Catalysis for Transesterification

Supporting Data 1

Porous Ti samples with a particle size of 45 μm (Zhongrui Material Technology Corp., Taiwan) were prepared *via* the P/M method using NaCl (Taiyen Biotech Corp., Taiwan) with particle sizes in the range of 180–300 μm as the space holder. NaCl was used as a permanent space holder because of its high solubility in water (359 g/L at room temperature), complete inertness with Ti powder. Mixtures containing Ti powder and 55 wt% NaCl (with respect to the weight of Ti powder) were compacted into a disc of 10 mm in diameter and 7 mm in thickness. The as-formed Ti samples were placed into an autoclave, which was also used as the container for the subsequent hydrothermal process. To remove NaCl completely before sintering, a temperature higher than 130 °C was applied to evaporate water. The as-heated Ti samples were dried at 80 °C in a cyclic oven (JA-27, Great Tide Instrument, Taiwan) for 24 hr to evaporate the remaining water. The solid Ti sample was evacuated three times under vacuum (10^{-3} mbar) and then sintered at 1000 °C for 3 hr. The as-sintered Ti sample is denoted as Ti_1000_0.

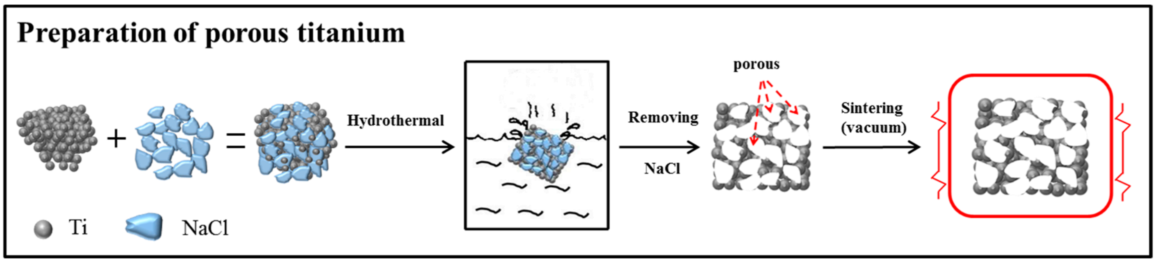

Figure S1. Fabrication processes: porous Ti samples are obtained by mixing Ti powder with NaCl, and followed by the hydrothermal technique, which may thereafter remove NaCl from the Ti matrix, leave the solid pore sites, and form porous structures.

Supporting Data 2

We have made a simple calculation about the grain sizes by XRD and the *Scherrer* formula (Equation 1); the average grain sizes of $Sr_{0.5}$_P_Ti-55 and $Sr_{0.7}$_P_Ti-55 were 25.9 and 43.1 nm, respectively.

Table S1. Calculation of grain size by using XRD.

Sample	K	λ (nm)	β	2θ	τ
$Sr_{0.5}$_P_Ti-55	0.89	0.15406	0.556	36	25.9
$Sr_{0.7}$_P_Ti-55	0.89	0.15406	0.334	36	43.1

$$\tau = \frac{K\lambda}{\beta cos\theta} \qquad (1)$$

Where,

τ - is the mean size of the ordered (crystalline) domains, which may be smaller or equal to the grain size.
K - is a dimensionless shape factor, with a value close to unity. The shape factor has a typical value of about 0.9, but varies with the actual shape of the crystallite
λ - is the X-ray wavelength.
β - is the line broadening at half the maximum intensity (FWHM), after subtracting the instrumental line broadening, in radians. This quantity is also sometimes denoted as $\Delta(2\theta)$.
θ - is the Bragg angle.
